# Combined Pulsed Electron Double Resonance EPR and Molecular Dynamics Investigations of Calmodulin Suggest Effects of Crowding Agents on Protein Structures

**DOI:** 10.1021/acs.biochem.2c00099

**Published:** 2022-08-18

**Authors:** Andrew M. Stewart, Muralidharan Shanmugam, Roger J. Kutta, Nigel S. Scrutton, Janet E. Lovett, Sam Hay

**Affiliations:** † The Roy J. Carver Department of Biochemistry, Biophysics and Molecular Biology, Iowa State University, Ames 50011, Iowa, United States; ‡ Manchester Institute of Biotechnology and Department of Chemistry, The University of Manchester, 131 Princess Street, Manchester M1 7DN, U.K.; § Institute of Physical and Theoretical Chemistry, University of Regensburg, Regensburg 93040, Germany; ∥ SUPA School of Physics and Astronomy and BSRC, The University of St Andrews, St Andrews KY16 9SS, U.K.

## Abstract

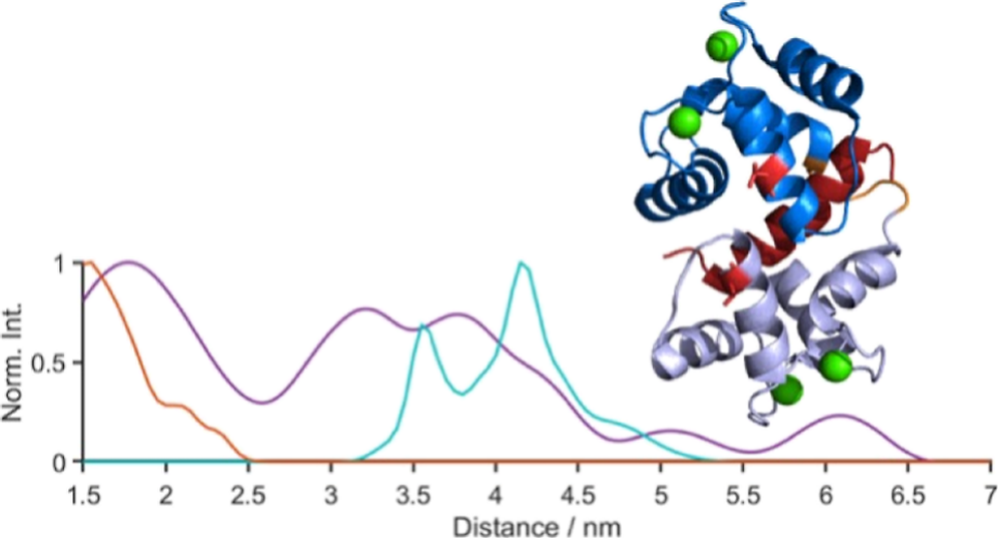

Calmodulin (CaM) is a highly dynamic Ca^2+^-binding protein that exhibits large conformational changes upon binding Ca^2+^ and target proteins. Although it is accepted that CaM exists in an equilibrium of conformational states in the absence of target protein, the physiological relevance of an elongated helical linker region in the Ca^2+^-replete form has been highly debated. In this study, we use PELDOR (pulsed electron–electron double resonance) EPR measurements of a doubly spin-labeled CaM variant to assess the conformational states of CaM in the apo-, Ca^2+^-bound, and Ca^2+^ plus target peptide-bound states. Our findings are consistent with a three-state conformational model of CaM, showing a semi-open apo-state, a highly extended Ca^2+^-replete state, and a compact target protein-bound state. Molecular dynamics simulations suggest that the presence of glycerol, and potentially other molecular crowding agents, has a profound effect on the relative stability of the different conformational states. Differing experimental conditions may explain the discrepancies in the literature regarding the observed conformational state(s) of CaM, and our PELDOR measurements show good evidence for an extended conformation of Ca^2+^-replete CaM similar to the one observed in early X-ray crystal structures.

## Introduction

Calmodulin (CaM) is a ubiquitous and promiscuous Ca^2+^-sensing protein. Binding to over 350 target proteins of diverse pathways,^1^ CaM helps regulate, both positively and negatively, a wide range of cellular functions.^2^ Although CaM binds the majority of its binding partners in a Ca^2+^-dependent manner, CaM is also capable of binding some proteins in a Ca^2+^-independent fashion.^3^ Although CaM does not recognize a consensus target sequence for binding, there are structural features on the target proteins which promote CaM binding.^4^ These include disordered helix-prone sequences with specifically spaced hydrophobic “anchors”, such as tryptophan residues.^5,6^ This plasticity in target recognition is the basis of the promiscuity of CaM.^7^ One problem with promiscuous binding proteins, and CaM in particular, is differential regulation when multiple targets are present in the same cell type. One hypothesis suggests that oscillating cellular Ca^2+^ and Mg^2+^ concentrations may mediate CaM binding, which is supported by the varied binding affinities for Ca^2+^ and Mg^2+^ at each of the four metal binding sites of the protein.^8^ However, this theory does not fully account for binding specificity, suggesting that other methods may also be employed.

**Figure 1:**
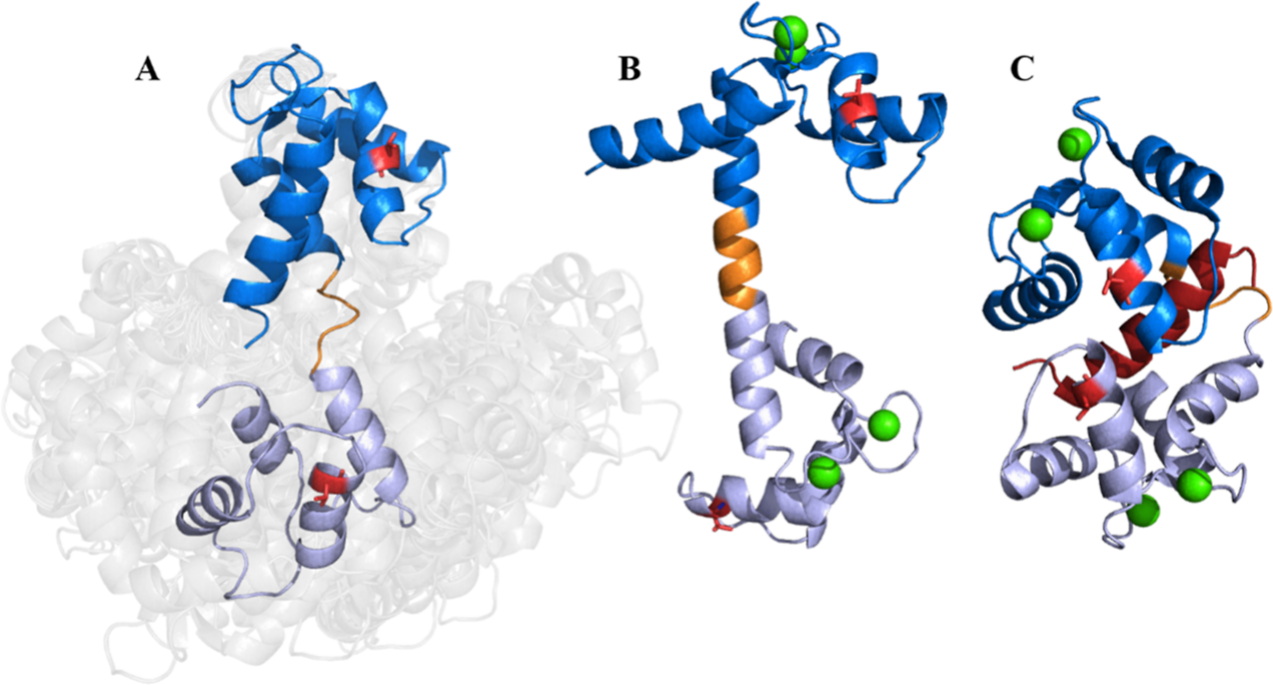
Published structures representative of the three major conformational states of CaM, color-coded by domain with the N-terminal domain in silver, the C-terminal domain in blue, the central linker region in orange, and Ca^2+^ ions in green. The MTSL labeling sites (38 and 110) used in this study are shown in red and the nNOS peptide in dark red in panel C. The NMR structures (PDB 1CFD and 1LKJ ensemble shown in gray) of apo-CaM are shown in (A), the X-ray crystal structure of CaM–Ca_4_^2+^ (PDB 1CLL) is shown in (B), and the X-ray crystal structure of CaM–Ca_4_^2+^ bound to an nNOS-derived CaM-binding peptide in red (PDB 2O60) is shown in (C).

CaM is a monomeric protein which consists of two homologous globular domains separated by a flexible linker. Each domain contains two EF-hand motifs, each of which is able to bind two Ca^2+^ atoms. When CaM binds its targets *via* the globular domains, it wraps around the target with the domains roughly opposing one another (the canonical binding conformation); therefore, an obvious mechanism for binding control is to modulate the conformation of the CaM inter-domain linker. The first high-resolution structure of CaM was solved by X-ray crystallography (Figure 1B); this structure showed Ca^2+^-replete CaM (four Ca^2+^ bound; denoted CaM–Ca_4_^2+^) with a highly elongated central helix, having the two terminal domains extended away from each other.^9^ This helix has subsequently been thought to be a crystallographic artifact due to the rarity of solvent-exposed helices of this length observed in other proteins and later observations by other techniques.^10^ In support of this hypothesis, the solution NMR structure of apo-CaM (Figure 1A), by contrast, showed CaM to be semi-elongated with the two domains being highly dynamic relative to one another.^11^ Additional studies by NMR^12,13^ and computation^14,15^ showed the central linker region of Ca^2+^-replete CaM to be highly flexible and not likely to form a stable elongated helix. Furthermore, in an attempt to study CaM by NMR while mimicking the crystal conditions, it was shown that CaM has the capacity to form a highly extended, but unstable helix in the presence of helix-inducing agents such as trifluoroethanol.^16^ This does not necessarily imply any physiological significance and was interpreted as evidence that the extended conformation seen in the crystal structure was stabilized by crystal contacts. However, it has been suggested that the changing cellular environment could impart similar conformational effects, leading to transient conformations which help CaM to modulate its protein binding affinities.^10^ With this in mind, it is of note that most CaM studies are performed with highly variable solvent conditions; specifically, the elongated crystal structure was determined in the presence of PEG 6000, while the Ca^2+^-replete NMR measurements were performed in the absence of any crowding agent. This is of importance, given the crowded nature of the cell. Collectively, the experimentally investigated structures of CaM suggest that an “*n*-state” model with *n* ≥ 3 is required to describe the conformational landscape. A three-state model would comprise (i) a semi-extended apo-CaM conformation with a highly disordered linker region and two terminal domains composed of four-helix bundles that are highly dynamic relative to each other, exemplified by the PDB structures 1CFD/1LKJ (Figure 1A), which encompass two of the many possible apo-CaM states,^17^ (ii) an extended Ca^2+^-replete conformation with the linker region extended in a helix separating the two domains. In the terminal domains, the Ca^2+^ ions are coordinated by four EF-hand motifs (two per domain); this coordination of Ca^2+^ destroys the four-helix bundles, exposing the hydrophobic core, exemplified by the PDB structure 1CLL (Figure 1B).^18^ (iii) The third state is less well-defined in that CaM is able to bind its targets by utilizing a number of conformations;^4^ however, the canonical binding conformation has CaM tightly clamped around a target helix, with large hydrophobic anchors in the hydrophobic cores of each domain, exemplified by the PDB structure 2O60 (Figure 1C).

In the current study, we use the EPR experiment of four-pulsed electron–electron double resonance (PELDOR) to derive distance measurements of CaM to determine whether the extended helix binding mode is present under physiologically relevant conditions.^19−22^ We also explore CaM binding to one of its protein targets, neuronal nitric oxide synthase (nNOS), in order to build a more complete picture of the CaM conformational landscape as it pertains to protein binding.^23^ Furthermore, we address the plausibility of CaM, utilizing an extended conformation in binding target proteins under specific conditions, and we highlight the importance of the solvent composition for such studies.

## Results

### Apo- and Ca^2+^-Bound CaM

**Figure 2:**
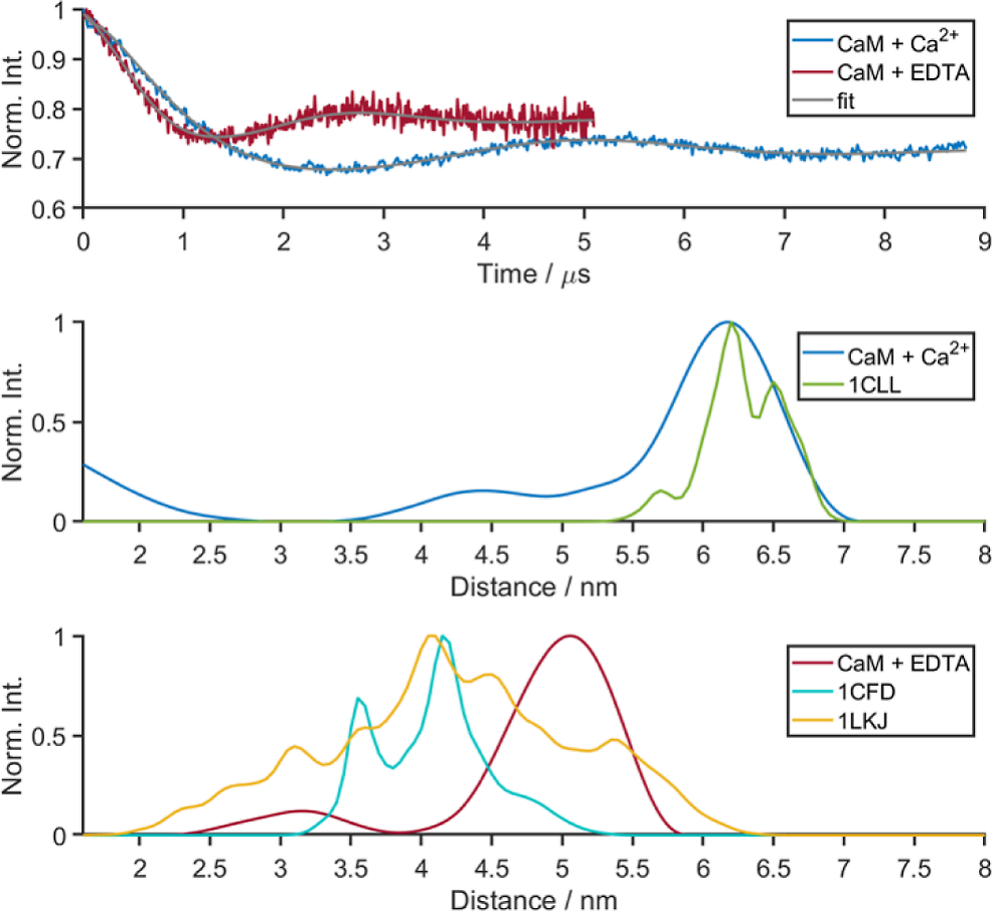
PELDOR time traces and derived distance distributions using DeerAnalysis 2021 of CaM + Ca^2+^ (blue) and CaM + EDTA (red, where EDTA is added to remove Ca^2+^ from the protein). Also shown are distance distributions derived using MMM from the published structures PDB IDs: 1CLL, 1CFD, and 1LKJ as described in the main text. The Supporting Information (Figure S1) presents the original time traces and validations of the distance distributions.

Because CaM does not have any native paramagnetic cofactors, site-directed spin labeling was employed using MTSL (a nitroxide spin label with a thiosulfonate ester functional group), which has been extensively used in spin labeling studies on a range of proteins.^21,24,25^ Since CaM also does not have any native Cys residues, the T34C/T110C variant of CaM was used for MTSL attachment (Figure 1). These mutations have been used extensively in fluorescence studies utilizing maleimide labels^26−32^ to monitor the conformational changes in CaM; CaM T110C labeled with MTSL has also been used to monitor CaM docking to nNOS by EPR,^33^ and recently, CaM T34C/T117C and CaM N53C/T110C were used by the Goldfarb group for *in vivo* PELDOR measurements.^34^ CaM T34C/T110C was doubly labeled with MTSL and is designated as CaM–MTSL_2_ in this study. Initially, PELDOR experiments were performed with CaM–MTSL_2_ in both the Ca^2+^-free apo-state (apo-CaM–MTSL_2_) and replete/saturated with Ca^2+^ (CaM–MTSL_2_–Ca_4_^2+^). Samples were measured at the Q band (34 GHz) in 50% glycerol-*d*_8_. Samples were prepared in deuterated buffer (50 mM HEPES pH 7.4 and 150 mM NaCl made up in D_2_O) to slow the spin relaxation time (Figure S1 in the Supporting Information) to allow for greater accuracy in measuring a broader range of distances by PELDOR.^35^ Distance distributions were extracted from the raw PELDOR time traces using DeerAnalysis^36^ (Figure 2). Both the apo-CaM–MTSL_2_ and CaM–MTSL_2_–Ca_4_^2+^ PELDOR time traces had modulation depths consistent with a good level of double spin labeling and intramolecular interactions and presented distinct modulations of the echo, which could be analyzed to give distance distributions. The apo-state distances were generally shorter than the Ca^2^-replete CaM (bottom panel in Figure 2). The apo-state average over the distance range was 4.80 nm with a standard deviation of 0.67 nm and the most probable distance of 5.06 nm. CaM–MTSL_2_–Ca_4_^2+^ data analysis provided an overall average of 5.45 nm with a standard deviation of 1.38 nm and a maximum probability at 6.17 nm. MMM^37,38^ was used to estimate the expected inter-spin label distance from the known CaM high-resolution structures, and the predicted distance distributions are shown in Figure 2. For CaM–MTSL_2_–Ca_4_^2+^, we used the published Ca^2+^-replete crystal structure for human CaM (1CLL). The MMM-calculated inter-MTSL distances for this structure predict nearly exactly the dominant observed distance measured experimentally. There is a considerable difference in the inter-MTSL distance of apo-CaM–MTSL_2_ using the two NMR structures (PDB 1CFD and 1LKJ), where 1CFD is a composite result and 1LKJ is an ensemble of structures, which were each theoretically labeled using MMM, and the results are combined in Figure 2. However, this difference also becomes arbitrary when considering the dynamic nature of this state as seen by observing the NMR ensemble (Figure 1A). Although this distance does not agree with either predicted distance, it does overlaps with the upper range of 1LKJ.

### CaM Binding to nNOS Peptide and Full-Length nNOS

**Figure 3:**
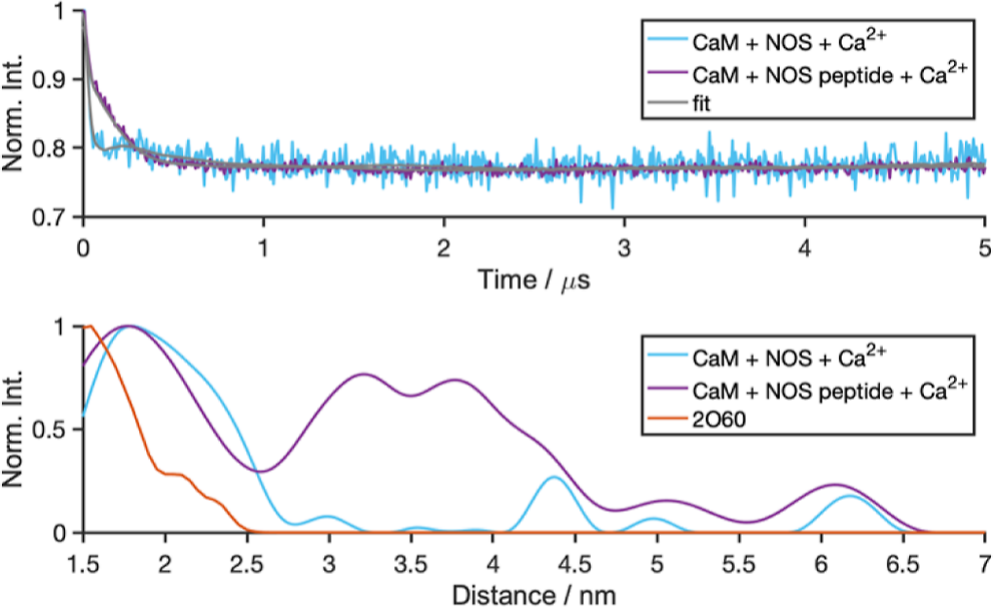
PELDOR time trace of CaM–Ca_4_^2+^ in the presence of nNOS (light blue) and the nNOS peptide (purple). Also shown are distance distributions derived using MMM from the published structures PDB ID: 2O60 as described in the main text. Additional data are shown in Figure S1 in the Supporting Information.

The conformational transitions from apo-CaM to CaM–Ca^2+^, while important, are ultimately regulatory states preparatory to binding of the target peptide. Over 300 proteins have been identified that bind to CaM. Although CaM is able to utilize various binding conformations, there is a set of related binding conformations which are considered canonical.^4^ In this study, nNOS was used to investigate the conformational states of CaM binding (Figures 3 and S1 in the Supporting Information) because it has been well-characterized and represents the canonical binding conformation of CaM. CaM–MTSL_2_ distances were measured by PELDOR in the presence of Ca^2+^ and either the CaM-binding peptide from nNOS (previously used to obtain the nNOS-bound CaM crystal structure PDB ID: 2O60) or full-length nNOS. Both sets of PELDOR measurements were performed with both a short and longer measurement window. The short measurement was used to better resolve the compact protein-bound conformation, while the longer measurement was used to observe any residual open (apo-CaM) or extended (CaM–Ca_4_^2+^) conformations. Both measurements are presented in Figure S1, and the longer time traces are shown and analyzed in Figure 3. In the presence of full-length nNOS, CaM exhibits a PELDOR time trace consistent with a short distance giving rise to a distance distribution predominantly at 1.8 nm, but it is also present with longer distances. The measurement with the peptide gives rise to a comparable short distance. The longer distances are now more predominant than in the presence of nNOS. The short distance seems consistent with the MMM prediction for the peptide-bound CaM using the PDB ID: 2O60 crystal structure in Figure 3. Although the longer-distance features may represent conformational states, where the peptide-bound CaM exhibits binding conformations not accessible in the presence of full-length NOS, it is unlikely that these longer distances represent CaM unbound to peptide as being Ca^2+^-replete, it should exhibit the ∼6 nm distance as seen above, and there is no change at this distance.

### Molecular Dynamics Simulations of CaM

**Figure 4:**
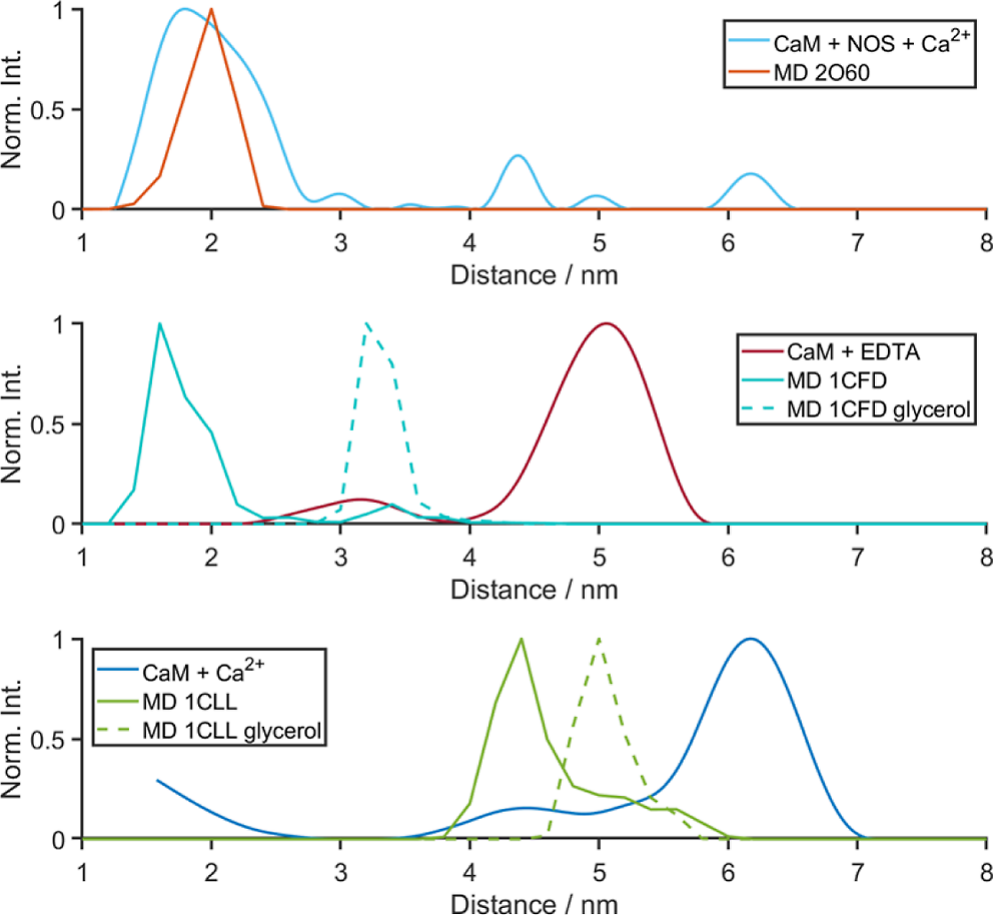
MD simulations of nNOS peptide-bound CaM–Ca_4_^2+^ (orange, 2O60), apo-CaM (red, 1CFD), and CaM–Ca_4_^2+^ (blue, 1CLL). The distribution of the inter-MTSL distance is plotted in each simulation, averaged over five replicates. Shown in solid lines are simulations in water (0% glycerol), and in dashed lines are simulations run in 30% v/v glycerol. Also shown are the PELDOR-derived distance distributions (CaM + NOS + Ca^2+^ in light blue, CaM + EDTA in red, and CaM–Ca_4_^2+^ in blue).

To further investigate the conformational sampling of CaM *in silico*, we utilized molecular dynamics (MD) simulations using the gromos54a7 force field modified with parameters for a MTSL-labeled cysteine (CYS) (parameters in Supporting Information). Starting structures used for MD simulations were the closed (nNOS peptide-bound) structure (PDB: 2O60), apo-CaM (PDB: 1CFD), and the Ca^2+^-replete structure (PDB: 1CLL). *In silico* mutagenesis was used to create the T34C/T110C variant with the Cys–MTSL residue at positions 34 and 110. All three structures were run for 100 ns with five replicates for a total of 500 ns for each structure (Figure 4). For comparison to the PELDOR data, all simulations were analyzed in terms of the inter-MTSL distance. The *in silico* peptide-bound structure did not change significantly from the initial crystal structure (Figure S2). However, both the apo-form and the Ca^2+^-replete form exhibited a substantial shortening of the inter-MTSL distance, relative to the PELDOR observations. This conformational change was the most pronounced in apo-CaM, where the protein collapsed into a compact conformation that superficially resembled the peptide-bound state (Figure S3); this result contrasts with solution NMR studies that found apo-CaM to be partially open. However, the loops containing T34C and T110C in simulations were further collapsed, occupying the space filled with the nNOS peptide in the 2O60 crystal structure. In the Ca^2+^-replete system, the shortened inter-MTSL distance was accounted for by the two terminal lobes moving in toward one another and folding in along the helix, while keeping both domains and the central helix structurally intact (Figure S4).

In an attempt to address the discrepancy between the conformations predicted by MD simulation and those observed in the PELDOR measurements, 30% glycerol v/v was added explicitly to the solvent in the MD simulations to mirror the experimental conditions (Figure S5). The Ca^2+^-bound structure and the apo form were re-run in the presence of this glycerol. The addition of glycerol did not fully recover the expected distances from the published structures of the PELDOR results, but the apo-CaM simulations (in the presence of glycerol) did retain the general semi-open conformation seen experimentally. We next wanted to assess any glycerol concentration dependence in the simulations and performed apo-CaM MD simulations with 5, 10, and 15% v/v glycerol (Figures 4 and S6). Any significant concentration dependence on glycerol did not appear *in silico* within the range (5–30%), but the presence of just 5% v/v glycerol appears sufficient to prevent CaM from adopting a collapsed bound-like conformation as in Figure S3. This saturation behavior is consistent with glycerol binding to or specifically interacting with the CaM protein. Indeed, during MD simulations, the glycerol appeared to cluster around the protein, which may explain, in part, why even 5% v/v glycerol was enough to prevent CaM from collapsing. These observations are in line with the observation that molecular crowders can affect the protein structure and conformational stability.^39−42^

## Discussion and Conclusions

CaM is a highly dynamic protein with hundreds of binding partners, all of which are differentially regulated by CaM under various cellular conditions. Despite being extensively studied, it is still not clear how CaM is able to specifically regulate so many protein targets. Although one level of control is likely to be in the various binding affinities of the four Ca^2+^ binding sites of CaM,^43^ the gross conformational state of CaM is also likely to play a role in the binding affinity to CaM’s binding partners. Numerous structural studies have provided conflicting information regarding the Ca^2+^-bound conformation, particularly with regard to the stability of the extended central helix. The major evidence for a highly extended helix is the initial crystal structure of CaM, although NMR and computational studies have highlighted the flexibility of the central region^11,44−46^ and argued against an extended helix. FRET studies of CaM conformational states have detected the presence of an extended state, comparable to the crystal structure by measure of inter-dye distance, although this was likely transient as it was only seen as a minor species which was not enhanced by the addition of Ca^2+^.^31^ Here, we attempt to resolve this disparity through EPR measurements of CaM under various conditions.

Surprisingly, our PELDOR data show a clear shift in the population equilibrium of the dominant species to a highly elongated conformation upon addition of Ca^2+^. Despite the seeming disparity with previous data, we suggest that the experimental conditions may be responsible for the observed populations. Our PELDOR measurements were performed in 50% glycerol at pH 7.4, while the FRET (pH 7.3) and NMR (pH 6.3) measurements did not have any added crowding agents or cryoprotectants. Since the MD results predicted that the glycerol needs to not be present and we require glycerol (or similar organic molecule) to act as a cryoprotectant and glassing agent, we were unable to measure PELDOR without it.

There have been computational studies using a course-grained approach looking at the effect of molecular crowding agents on the conformational state of CaM, and these suggested that Ficoll (modeled as a large rigid sphere) promoted a collapsed conformation.^47^ This is the opposite behavior to that observed in our studies, which found the presence of glycerol in the MD simulations to support a more open conformation. However, the same group did a FRET study of CaM with 10 or 20% Ficoll-70, Dextran-10, and sucrose (labeling T34/110C with Alexa 488 and QSY9) and found that while the cosolutes did promote a more compact state, the inter-label distance was still ∼50 Å (a reduction of ∼10 Å).^48^ In another study, looking at pharmaceutical protein GA-Z using asymmetric field-flow fractionation and small-angle neutron scattering (an 11 kDa protein with two domains connected by a flexible linker), Ramm *et al.* found that increasing glycerol concentrations favored compact domains with an overall elongated conformation, agreeing with our MD results.^42^ These observations suggest that although all the above cosolutes are compacting CaM, the relative smaller size of glycerol may allow it to penetrate between the N- and C-terminal domains of CaM, compacting the domains separately and effectively supporting a more elongated structure, while the larger cosolutes are acting on CaM wholly, promoting full-length CaM compaction.

In addition to solvent effects on CaM conformations, our data also suggests that the presence of other proteins, including non-binding domains of CaM targets, may affect the conformations accessible to CaM as in the case of binding to full-length NOS, which appears to restrict the number of binding conformations allowed by CaM relative to binding the NOS peptide alone. A recent study by the Goldfarb group investigated the conformational states of Gd-labeled CaM by W-band EPR *in vivo*.^34^ A direct comparison of the observed distances in not possible both because the labeling sites are not the same (T34C/T117C and N53C/T110C instead of T34C/T110C) and because the Gd label used has a longer linker than that of the nitroxide label used in this study. Considering these differences, the trends seen *in vivo* agree with the data presented in the present study, namely, a three-state model for CaM. Interestingly, the measurements performed in cells showed a significantly different behavior than that observed *in vitro* and cell extract. The authors note that the difference between in cell and cell extract is likely due to partner proteins made available by the cell extract process. Although this is possible, our results suggest that the conformational states may also be affected by the change in solvent conditions going from the ordered cellular environment to the more disordered extract. Very recently, a study by the Clore group investigated the time-resolved binding of CaM to a peptide derived from myosin light-chain kinase using DEER. Although also not directly comparable due to labeling sites used (S17C/A128C), the final peptide-bound state they measured is broadly consistent with our observations.^49^ Another study by the Goldfarb group compared DEER and NMR measurements of CaM binding to two peptides (IQ and MARCKS), representing two different binding conformations of CaM, both of which have corresponding crystal structures of CaM bound to the respective peptide.^50^ It was found that solution measurements of CaM bound to the IQ peptide were consistent with the crystal structure, but with the MARCKS peptide, there were significant discrepancies. However, when considering the difference between CaM binding to full-length NOS and the NOS peptide in our work, this discrepancy may be due to the lack of flanking protein structures which likely affect CaM conformations, which may be compensated for in the crystal structure by crystal packing. In another recent study, an increase in the intraprotein interdomain electron transfer (ET) rate in the bidomain oxygenase/FMN and the holoprotein of human inducible NOS was observed upon addition of Ficoll 70. This was interpreted in terms of an excluded volume effect, with an entropic origin,^51^ and the increased rate of ET would suggest that a more compact conformation with a shorter ET distance is preferred in the presence of this crowding agent. In future work, it would be interesting to investigate the effect of common cellular proteins and/or crowding agents on the conformational states of CaM at room temperature using NMR, FRET, or other methods in order to further elucidate how various cellular environments may modulate CaM affinity toward its binding partners. Our results indicate that an elongated central helix is likely present in CaM under certain conditions, suggesting that the PDB: 1CLL X-ray structure is not artifactual but instead captures a conformation of CaM that may be stabilized by specific cellular factors and is part of the binding regulation mechanism of this dynamic protein.

## Experimental Section

### Protein Production

The T34C T110C variant of CaM from *Gallus gallus* (UniProtKB—P62149), which is 100% identical to human CaM, was produced recombinantly in *Escherichia coli* and purified essentially as described previously using a phenyl-Sepharose column.^52^ The purified CaM was dialyzed in 40 mM HEPES, pH 7.6, 150 mM NaCl, and 1 mM CaCl_2_ and concentrated *via* centrifugal concentration (10 kDa MWCO) to a final concentration of ∼800 μM. Concentration was determined using UV–vis spectroscopy using ε_277nm_ = 3.03 mM^–1^ cm^–1^. His-tagged nNOS from *Rattus norvegicus* (UniProtKB—P29476) was produced recombinantly in *E. coli* and purified essentially as described previously using a Ni-Sepharose (Ni-NTA) column.^53^ The purified nNOS was dialyzed in 40 mM HEPES pH 7.6, 10% glycerol, 150 mM NaCl, 2.5 mM dithiothreitol (DTT), and 2 μM tetrahydrobiopterin (H4B). Sodium dithionate-reduced CO-bound nNOS was prepared to determine protein concentration using ε_444nm_ = 74 mM^–1^ cm^–1^. Activity data are given in Figure S7 in the Supporting Information.

### nNOS Peptide

The CaM binding peptide from nNOS, AIGFKKLAEAVKFSAKLMGQ, was purchased from GenScript. Stock solutions were prepared by weight and made up in sample buffer (40 mM HEPES, pH 7.6, and 150 mM NaCl).

### CaM Spin Labeling

CaM was spin-labeled with MTSL (Toronto Research Chemicals). DTT was added to 4 mL of CaM (100 μM) for a final concentration of 5 mM DTT and left on ice for 60 min. The DTT was removed from the CaM solution *via* an Econo-Pac DG10 desalting column (Bio-Rad). MTSL dissolved in 50 μL of MeOH was added to CaM solution to give a final concentration of 1 mM MTSL and incubated at 25 °C. Excess spin label was removed *via* a desalting column.

### PELDOR Experiments

EPR samples were prepared with 20 μM CaM and 20 μM nNOS or 200 μM peptide (when used) in 40 mM HEPES, pH 7.6, 150 mM NaCl, deuterium oxide, and 50% glycerol-*d*_8_. Sample buffer also contained 1 mM CaCl_2_ or 10 mM EDTA (when specified). EPR was carried out using a Bruker ELEXSYS E580 system at the Q band (34 GHz) with a cryogen-free variable temperature cryostat from Cryogenic Ltd., a 150 W TWT amplifier, and an EN 5106QT-2w cylindrical resonator. Experiments were carried out at 50 K using the four-pulse PELDOR experiment (refs (19) and (22)). A pulspel script was used to control the experiment. Two-step phase cycling was used with the time between the first two pulses being stepped five times with an initial value of 200 or 400 ns, with a delay increment of 24 ns to average out any unwanted hyperfine coupling effects. The exception was for the CaM with EDTA, which did not have the τ-averaging applied. The pump pulse was incremented by 8 ns, with exception for replete CaM which used a 16 ns time increment. The pulse lengths were 32 ns for the observer sequence, and the pump pulse was 14 ns. The shot repetition time was set to collect approximately 80% of the recovered echo. DeerAnalysis 2021 in the expert mode was used with Tikhonov regularization to provide distance distributions.^36^ The Supporting Information (Figure S1) gives further information of the individual parameters for the PELDOR experiment and the analysis.

### Computational Modeling and Molecular Dynamics

MMM^37,38^ was used to calculate rotamer populations and predict PELDOR distance distributions for 1cfd, 1cll, 1lkj, and 2o60.pdb files.

MD simulations were performed in GROMACS 5.0.4 using a modified gromos54a7 force field with parameters for CYS-conjugated MTSL (CYSa) and glycerol. Mutagenesis *in silico* was performed by aligning CYSa with either T34 or T110 using the PyMOL align protocol to attain coordinates, and TYR was then manually replaced with CYSa in the PDB file. All simulations were run with explicit solvent, under neutral conditions by adding NaCl to the simulation, and were energy-minimized and equilibrated for 100 ps with *NVT* (constant number of particles, volume, and temperature) and *NPT* (constant number of particles, pressure, and temperature) ensembles to stabilize the temperature and the pressure, respectively, followed by 100 ns MD simulation; each simulation was run five times. Simulations were performed in a 10 nm cubic water box. Glycerol simulations were run using the same procedure, except glycerol was inserted into the system prior to solvation.

### MD Parameterization

CYS-conjugated MTSL was parameterized for MD simulations by fusing the structures of MTSL and CYS. Parameters are given in the Supporting Information. Missing parameters were taken from matching pre-defined parameters in the GROMOS 54a7 force field, and charges were adapted in accordance to a UHF-MP2 PCM calculation in Firefly 8.0.0^54^ [which is partially based on the GAMESS (US)^55^ source code] using the TZV basis set. Glycerol parameters for use with the GROMOS 54a7 force field was acquired from the Automatic Topology Builder (ATB)^56^ (ATB molid: 29325, ATB Topology Hash: a8963). Final topology generation was performed using a B3LYP/6-31G*-optimized geometry, bonded and van der Waals parameters were taken from the GROMOS 54A7 parameter set, and initial charges were estimated using the ESP method of Merz–Kollman.^57^ Final charges and charge groups were generated by the method described in the ATB paper.^56^ Additional bonded parameters were generated from a Hessian matrix calculated at the B3LYP/6-31G*^58^ level of theory.

## Supporting Information Available

Additional methodological information, EPR and MD simulation data, nNOS activity data, and MD parameters (PDF)

## Accession Codes

CaM, P62149. nNOS, P29476.
