# Supplementary material for: Combined Pulsed Electron Double Resonance EPR and Molecular Dynamics Investigations of Calmodulin Suggest Effects of Crowding Agents on Protein Structures
Source: Biochemistry. 2022 Aug 18;61(17):1735–42. doi: 10.1021/acs.biochem.2c00099 (PMC9454100; doi:10.1021/acs.biochem.2c00099)
Supplement: Supplementary file 1 — bi2c00099_si_001.pdf [file bi2c00099_si_001.pdf]

Supporting Information:

Combined pulsed electron double resonance EPR and molecular dynamics investigations of calmodulin suggest effect of crowding agents on protein structure

Andrew M. Stewart,[a,b] Muralidharan Shanmugam,[b] Roger J. Kutta,[b,c] Nigel S. Scrutton,[b] Janet E. Lovett,[d*] Sam Hay[b*]

[a] The Roy J. Carver Department of Biochemistry, Biophysics and Molecular Biology, Iowa State University, Ames 50011, IA, USA.

[b] Manchester Institute of Biotechnology and Department of Chemistry, The University of Manchester, 131 Princess St, Manchester M1 7DN, UK

[c] Institute of Physical and Theoretical Chemistry, University of Regensburg, 93040 Regensburg, Germany

[d] SUPA School of Physics and Astronomy and BSRC, The University of St Andrews, St Andrews, KY16 9SS, UK

*Corresponding authors:
sam.hay@manchester.ac.uk
jel20@st-andrews.ac.uk

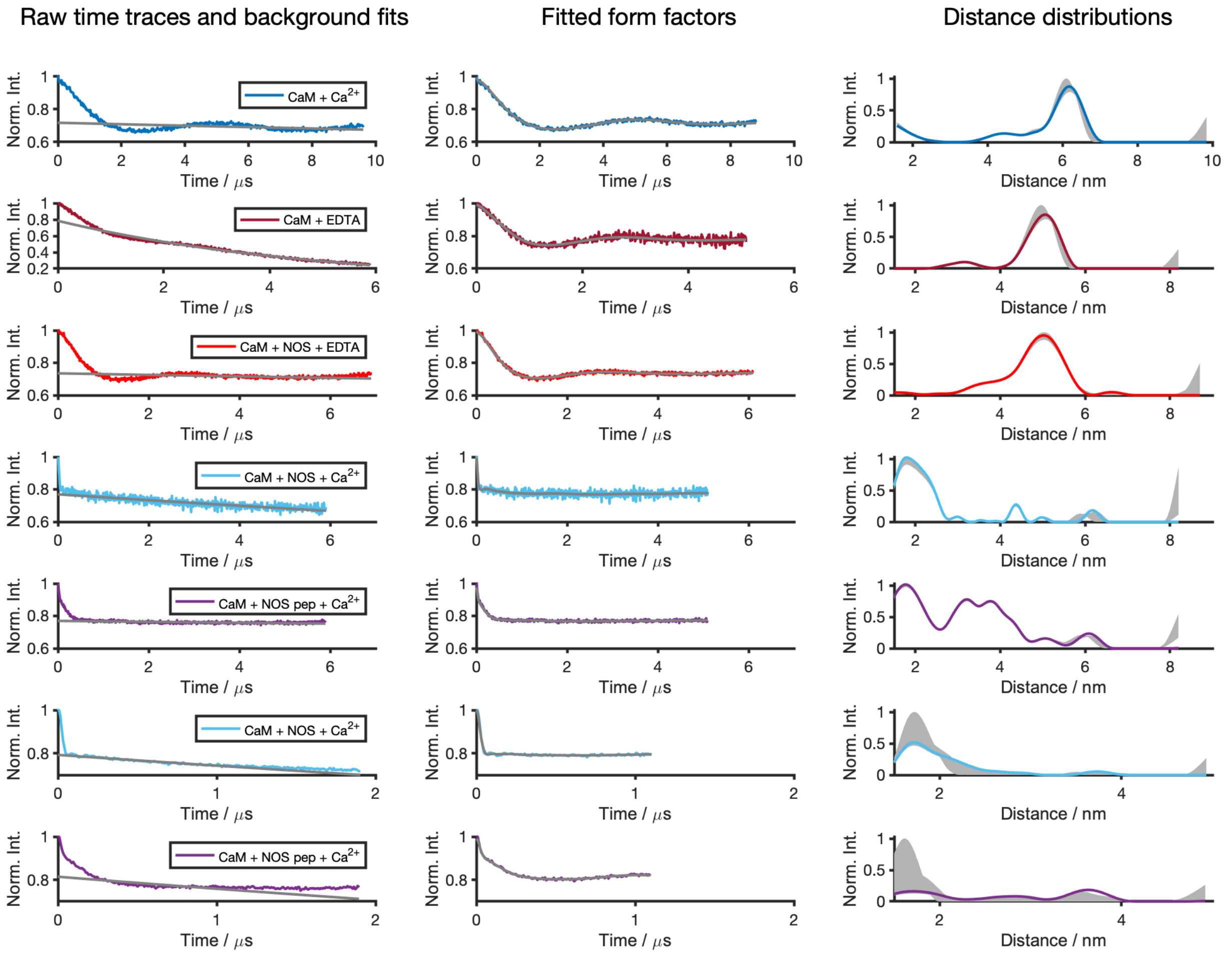

Figure S1: Experimental PELDOR time traces of all measurements (left) shown with background correction (middle) and derived distance distributions (right). Data were processed using DeerAnalysis2021 and Tikhonov Regularization. The grey line for the left panel shows the fitted background, for the middle panel it shows the distance distribution fit and for the right the best fit distance is given in color and a measure of the certainty is given in grey using a validation procedure. Data on rows 1,2, 4 and 5 are shown in the main paper but here includes validation. The short time trace data in rows 6 and 7 are to support the conclusions of the analysis. The data on row 3 are for apo-CaM with NOS present which demonstrate that CaM does not appear to bind NOS without the presence of calcium.

DeerAnalysis2021 in Matlab r2021a on Windows10 was used. Though used in expert mode, the "!" automatic calculations were used except that all data were cut from the end by 800 ns to avoid any artefacts that can manifest at the end of the data. Hence the Form Factors (background corrected data) in the figure are shorter than the experimental time traces. "Noisy" was checked. The L-curve method was used to calculate the Tikhonov Regularization parameter and the program-calculated one was used. Validation used the default values for background correction start points and 11 steps. Further, experimental details in addition to those given in the main paper: **Row 1** (replete CaM) was measured with a decay between the first and second PELDOR pulse (d1) of 400 ns, a delay between the first echo and the third observer pulse (d2) of 10000 ns and 51 averages were collected. The deadtime during which no data were collected was 380 ns, whereas this is 80 ns for other data sets. DeerAnalysis: zero-time 37 ns; background start value 3040 ns;

phase -6.2; Tikhonov Regularization parameter 1580. Modulation depth 0.282; average 5.45 nm and sigma 1.38 nm. Validation background steps 1920 to 5776 ns.

Row 2 (apo CaM) used d1 = 200 ns and d2 = 6000 ns. 95 averages were collected (no tau-averaging). DeerAnalysis: zerotime 135 ns; background start value 1616 ns; phase -2.4; Tikhonov Regularization parameter 3980. Modulation depth 0.208; average 4.80 nm and sigma 0.67 nm. Validation background steps 1176 to 3536 ns.

Row 3 (apo CaM with NOS present) used d1 = 400 ns and d2 = 7000 ns. 15 averages were collected. DeerAnalysis: zerotime 324 ns; background start value 3184 ns; phase -5.4; Tikhonov Regularization parameter 1260. Modulation depth 0.286; average 5.47 nm and sigma 1.42 nm. Validation background steps 1384 to 4144 ns.

Row 4 (CaM with calcium and NOS) used d1 = 400 ns and d2 = 6000 ns and 6 averages. DeerAnalysis: zerotime 328 ns; background start value 512 ns; phase -2.1; Tikhonov Regularization parameter 794. Modulation depth 0.150; average 2.53 nm and sigma 1.23 nm. Validation background steps 1184 to 3544 ns.

Row 5 (CaM with calcium and NOS peptide) used d1 = 200 ns and d2 = 6000 ns and 64 averages. DeerAnalysis: zerotime 130 ns; background start value 504 ns; phase -9.0; Tikhonov Regularization parameter 1260. Modulation depth 0.225; average 3.21 nm and sigma 1.23 nm. Validation background steps 1176 to 3536 ns.

Row 6 (CaM with calcium and NOS) used d1 = 400 ns and d2 = 2000 ns and 8 averages. DeerAnalysis: zerotime 327 ns; background start value 184 ns; phase -0.5; Tikhonov Regularization parameter 25.1. Modulation depth 0.120; average 2.04 nm and sigma 0.5 nm. Validation background steps 384 to 1144 ns.

Row 7 (CaM with calcium and NOS peptide) used d1 = 200 ns and d2 = 2000 ns and 8 averages. DeerAnalysis: zerotime 129 ns; background start value 160 ns; phase 2.3; Tikhonov Regularization parameter 50.1. Modulation depth 0.176; average 2.75 nm and sigma 0.83 nm. Validation background steps 376 to 1136 ns.

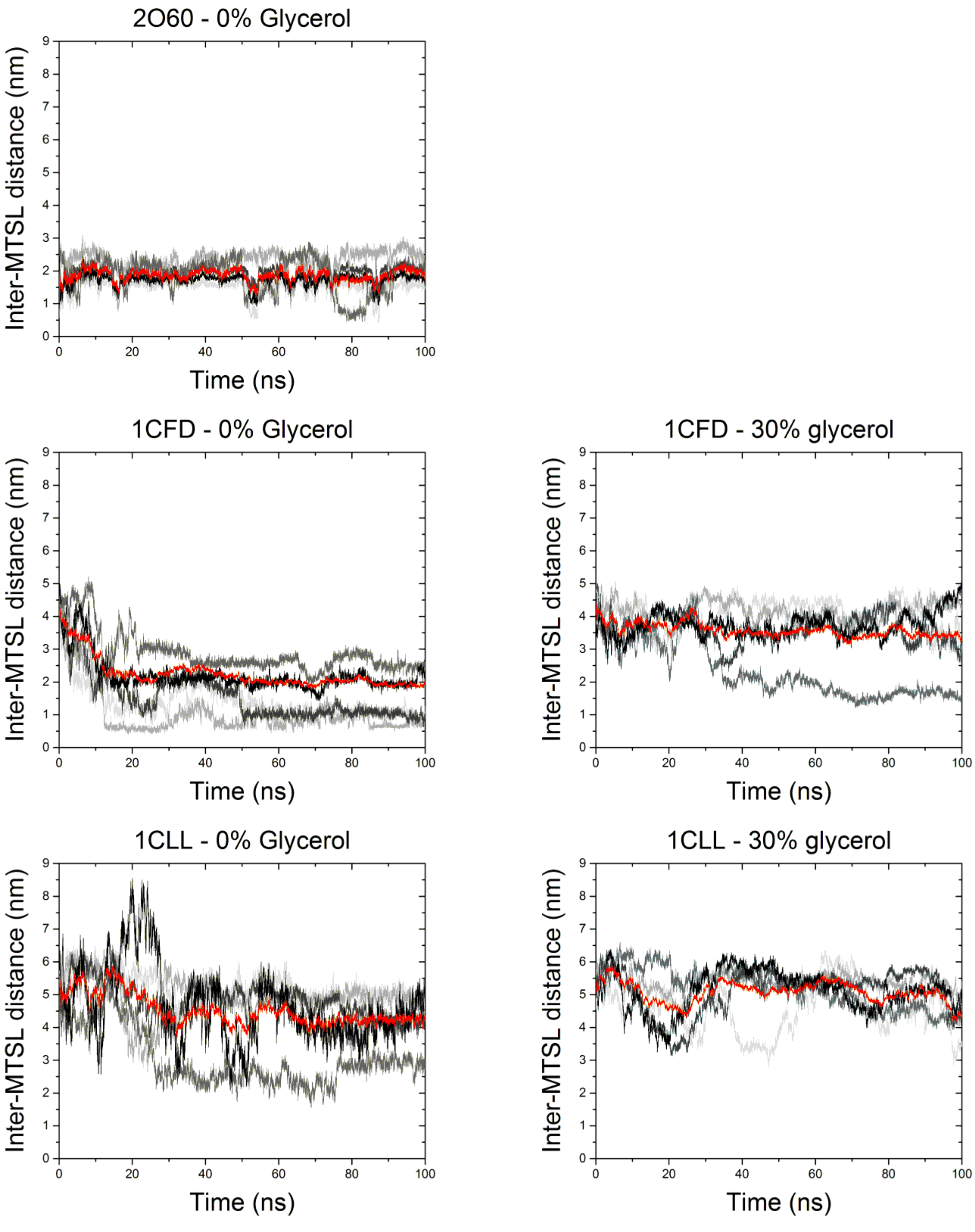

Figure S2: MD simulations of nNOS peptide bound CaM (2O60), apo-CaM (1CFD) and CaM-4Ca^{2+} (1CLL). Graphs show the distance between two MTSL spin labels at the T34C and T110C positions plotted against simulation time (100 ns). Shown on the left are simulations in water (0% glycerol), on the right are simulations run in 30% glycerol. Individual replicants shown in monochrome gradient, average of all replicants shown in red.

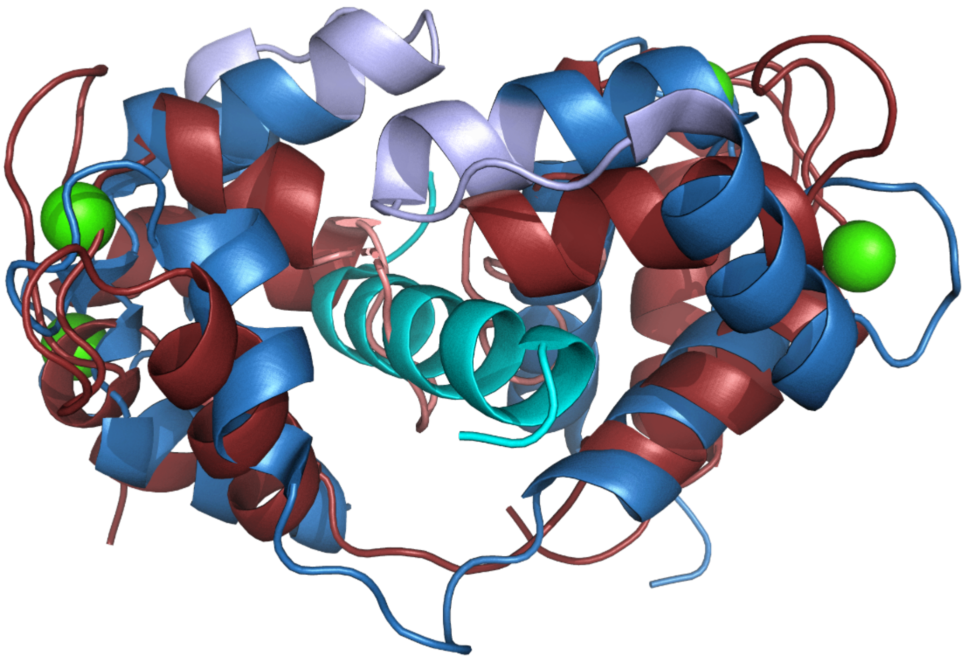

Figure S3: Representative final structure from the 1CFD MD simulations (red/salmon) shown with the crystal structure, 2O60, of CaM (blue/light blue) bound to the nNOS peptide (cyan). Corresponding loop regions are shown in light blue and salmon to highlight the conformational difference of these two structures, notably that the loops in the 1CFD MD simulations occupy the space filled by the nNOS peptide in the crystal structure.

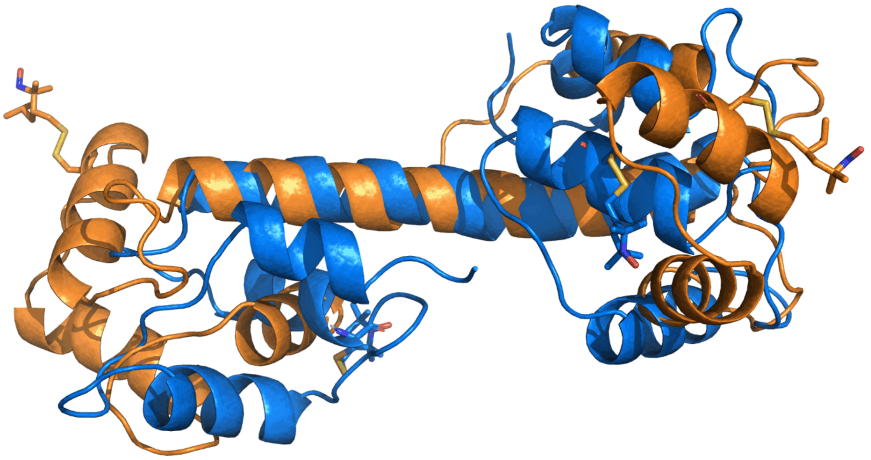

Figure S4: Representative structures of initial (orange) and final (blue) states from the 1CLL MD simulations showing the terminal lobes pulling in around the central helix.

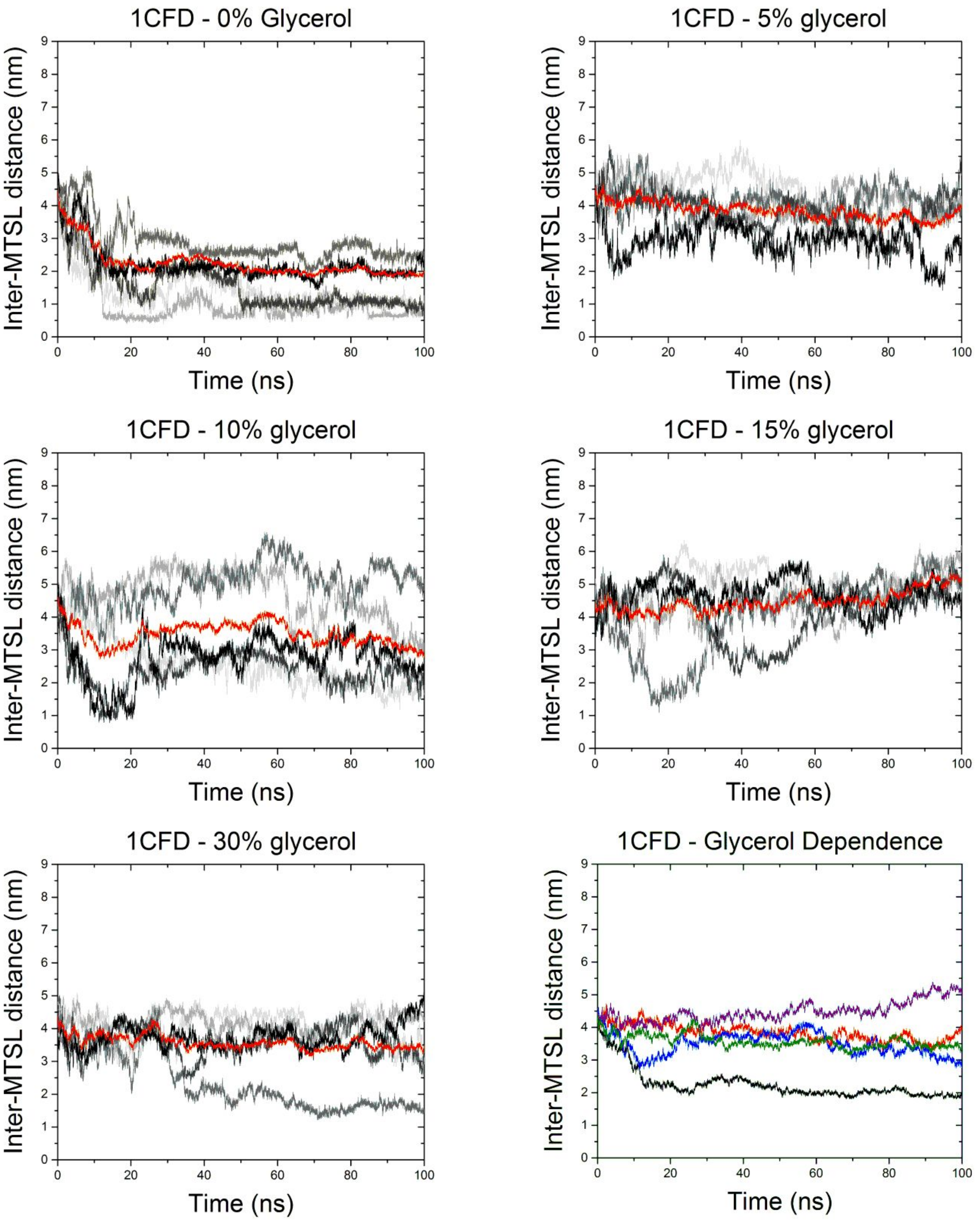

Figure S5: MD simulations of apo-CaM (1CFD) in varying amounts of glycerol. (all except bottom-right) individual replicants shown in monochrome gradient, average of replicants shown in red. (bottom-right) all averages of 1CFD glycerol simulations with 0% (black), 5% (red), 10% (blue), 15% (purple) and 30% (green). Distances correspond to CaM T34/110C-MTSL distances.

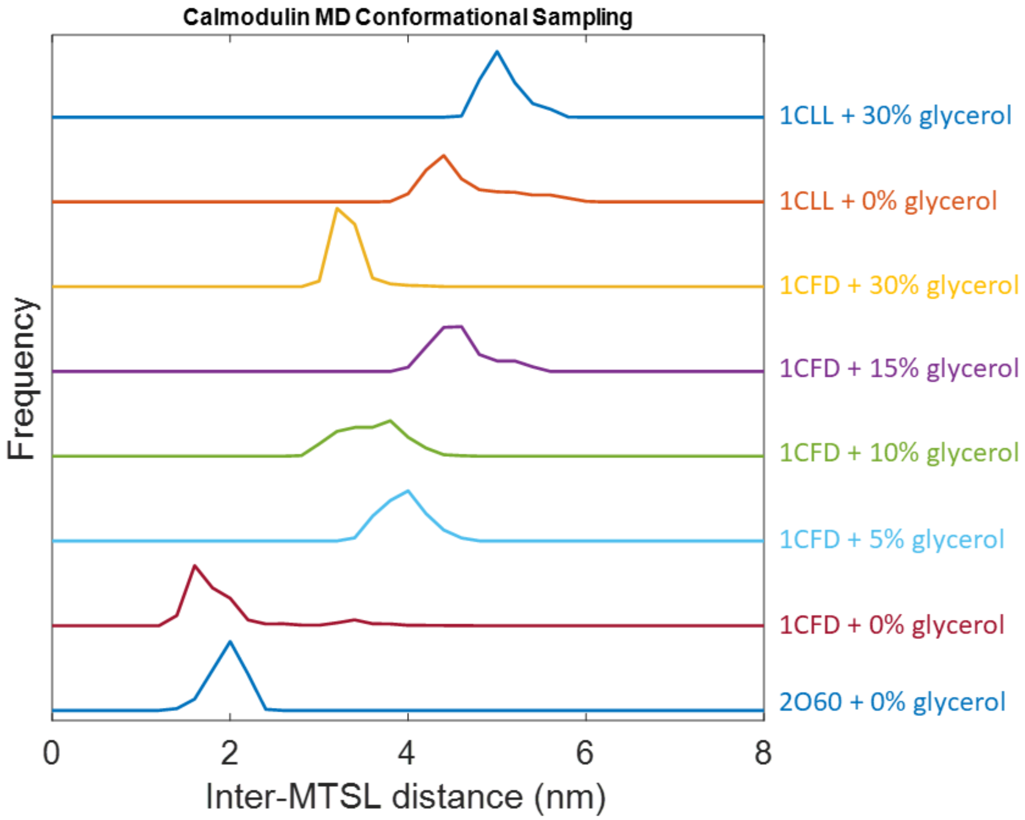

Figure S6: Distance distribution results from the MD simulations of apo-CaM (1CFD) in varying amounts of glycerol. Vertical lines represent MTSLwizard distances derived from published structures for CaM bound to the nNOS peptide (2O60; black), apoCaM (1CFD; red), and CaM-4Ca^{2+} (1CLL; blue). Distances correspond to CaM T34/110C-MTSL distances.

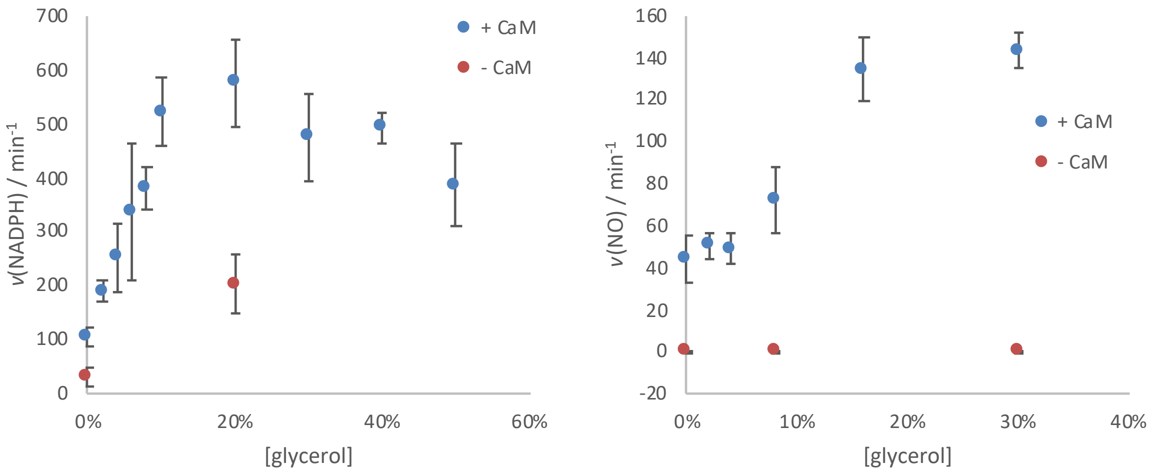

Figure S7: The effect of glycerol on nNOS activity. NADPH oxidation (*left*) and NO production (*right*) was measured with wt-nNOS and wt-CaM (blue) or wt-nNOS in the absence of CaM (red) at 25 ^{o}C. The initial rate of NADPH oxidation was measured spectroscopically at 340 nm using an extinction coefficient for NADPH of 6.22 mM^{-1} cm^{-1}. Samples contained 25 nM nNOS, 40 mM HEPES, pH 7.6, 15 mg/ml CaM, 1 mM $CaCl_2$, 0.3 mM DTT, 5 mM Arg, 4 mM each of FAD and H_4B. The reaction was initiated by adding NADPH to the reaction mixture to a final concentration of 0.2 mM. The initial rate of NO synthesis was measured using the oxyhemoglobin assay for NO. The reaction was monitored spectroscopically at 401 nm using an extinction coefficient for oxyhemoglobin of 38 mM^{-1} cm^{-1}. The nNOS (25 nM) was added to a cuvette containing 15 mg/ml CaM, 0.62 mM $CaCl_2$, 0.3 mM DTT, 5 mM Arg, 4 mM each of FAD and H_4B, and 10 mM oxyhemoglobin in 40 mM HEPES, pH 7.6 to give a final volume of 0.7 ml. The reaction was started by adding NADPH to give a final concentration of 0.2 mM.

Is nNOS native-like in high glycerol?

We have previously reported PELDR spectra of the disemiquinoid (2-electron reduced) state of nNOS in 30% glycerol.[1] A number of conformations with different inter-flavin distances were observed, and perturbation of the PELDR spectrum was observed upon addition of CaM, consistent with CaM binding to nNOS (both reductase domain and full-length protein) under these conditions. In the present study, we measured a rate of NO production at 0% glycerol of 44 ± 11 min^{-1}, which is comparable to previous reports (k_{cat} = 50-100 min^{-1}; Table 1 in ref *2*) and a rate of NADPH oxidation of 104 ± 18 min^{-1} (**Figure S7**). At 30% glycerol we observe a 3.2 ± 0.8-fold increase in the rate of NO production relative to 0% glycerol. The rate of NADPH consumption is also enhanced by the addition of glycerol, suggesting that uncoupling (non-productive oxidation of NADPH) becomes more prevalent under these conditions. Together, these data demonstrate that nNOS is still active under in 30% glycerol (i.e. the active site is intact and functional), but there is some perturbation to the nNOS-CaM complex that enhances the rate of NADPH oxidation and NO production, but also promotes uncoupling. These changes in activity could involve glycerol-induced conformational change, and/or perturbation of the electrostatic control of substrate binding and/or inter-domain electron transfer.[e.g. 3, 4]

MTSL MD Parameters:

;CYS-MTSL GROMOS parameters.
;CYSa was created by fusing CYS with MTLS.
;The missing forcefield parameter are taken from matching pre-defined
;parameter in the GROMOS forcefield and the charges were adapted
;in accordance to a UHF-MP2_PCM(water)_TZV calculation

```
[ CYSa ]
[ atoms ]
N  N -0.28000  0
H  H  0.28000  0
CA CH1  0.00000  1
CB CH2  0.05000  2
SG  S -0.03800  2
C  C  0.38000  3
O  O -0.38000  3
S1  S -0.03500  2
C1 CH2 -0.13400  2
C2a  C  0.09400  4
C2b CR1  0.08700  4
C3a  C  0.13500  4
CMa1 CH3  0.03900  4
CMa2 CH3  0.03900  4
C3b  C  0.13400  4
CMb1 CH3  0.05200  4
CMb2 CH3  0.05200  4
N1 NL -0.06900  4
ON OA -0.40600  4
[ bonds ]
N  H gb_2
N CA gb_20
CA  C gb_26
C  O gb_4
C +N gb_9
CA CB gb_26
CB SG gb_30
SG S1 gb_a7
S1 C1 gb_a6
C1 C2a gb_a1
C2a C2b gb_a5
C2a C3a gb_a3
C3a CMa1 gb_a1
C3a CMa2 gb_a1
C2b C3b gb_a3
C3b CMb1 gb_a1
C3b CMb2 gb_a1
```

```
 C3a N1 gb_a2
 C3b N1 gb_a2
 N1 ON gb_a4
;[ pairs ]
; CB C1
; SG C2a
; S1 C2b
; C2a CMb2
; C2a CMb1
; C1 C3b
; CMa1 ON
; CMa1 C3b
; CMa1 C2b
; CMa1 C1
; C3a CMb1
; C3a CMb2
; C3a S1
; CMa2 ON
; CMa2 C3b
; CMa2 C2b
; CMa2 C1
; N1 C1
; ON CMb1
; ON CMb2
; ON C2b
; ON C2a
 [ angles ]
; ai aj ak gromos type
 -C  N  H  ga_31
 H  N CA  ga_17
 -C  N CA  ga_30
 N CA  C  ga_12
 CA  C +N  ga_18
 CA  C  O  ga_29
 O  C +N  ga_32
 N CA CB  ga_12
 C CA CB  ga_12
 CA CB SG  ga_15
 CB SG S1  ga_a11
 SG S1 C1  ga_a10
 S1 C1 C2a  ga_a9
 C1 C2a C2b  ga_a8
 C1 C2a C3a  ga_a8
 C2b C2a C3a  ga_a7
 C2a C2b C3b  ga_a7
 C2b C3b CMb2  ga_a6
```

C2b C3b CMb1 ga_a6
CMb2 C3b CMb1 ga_a1
C2b C3b N1 ga_a3
CMb2 C3b N1 ga_a2
CMb1 C3b N1 ga_a2
C3b N1 ON ga_a5
C3b N1 C3a ga_a3
ON N1 C3a ga_a4
C2a C3a N1 ga_a3
C2a C3a CMa2 ga_a1
N1 C3a CMa2 ga_a2
C2a C3a CMa1 ga_a1
N1 C3a CMa1 ga_a2
CMa2 C3a CMa1 ga_a1
[impropers]
; ai aj ak al gromos type
N -C CA H gi_1
C CA +N O gi_1
CA N C CB gi_2
CMa2 N1 CMa1 C3a gi_2
C3b ON C3a N1 gi_2
CMb2 CMb1 N1 C3b gi_2
C2b C1 C3a C2a gi_1
[dihedrals]
; ai aj ak al gromos type
-CA -C N CA gd_4
-C N CA C gd_19
N CA C +N gd_20
N CA CB SG gd_17
C1 S1 SG CB gd_10
SG S1 C1 C2a gd_13
C3a C2a C1 S1 gd_20
C3b C2b C2a C1 gd_5
C2a C2b C3b N1 gd_20
C3a N1 C3b C2b gd_14
C1 C2a C3a CMa1 gd_20
C3b N1 C3a CMa1 gd_14

Additional References.

[1] Sobolewska-Stawiarz, A., Leferink, N. G. H., Fisher, K., Heyes, D. J., Hay, S., Rigby, S. E. J., and Scrutton, N. S. (2014) Energy Landscapes and Catalysis in Nitric-oxide Synthase, *J. Biol. Chem. 289*, 11725-11738.

[2] Leferink, N. G. H., Hay, S., Rigby, S. E. J., and Scrutton, N. S. (2015) Towards the free energy landscape for catalysis in mammalian nitric oxide synthases, *FEBS J. 282*, 3016-3029.

[3] Schrammel, A., Gorren, A. C. F., Stuehr, D. J., Schmidt, K., and Mayer, B. (1998) Isoform-specific effects of salts on nitric oxide synthase activity, *Biochim. Biophy. Acta 1387*, 257-263.

[4] Feng, C., Chen, L., Li, W., Elmore, B. O., Fan, W., and Sun, X. (2014) Dissecting regulation mechanism of the FMN to heme interdomain electron transfer in nitric oxide synthases, *J. Inorg. Biochem.130*, 130-140.
